# Telomere sister chromatid exchange and the process of aging

**DOI:** 10.18632/aging.100206

**Published:** 2010-09-23

**Authors:** Krastan B. Blagoev, Edwin H. Goodwin, Susan M. Bailey

**Affiliations:** ^1^ National Science Foundation, Arlington, VA 22230, USA; ^2^ KromaTiD Inc., Fort Collins, CO 80524, USA; ^3^ Department of Environmental and Radiological Health Sciences, Colorado State University, Fort Collins, CO 80523-1618, USA

**Keywords:** T-SCE, aging, WRN, BLM

## Abstract

Telomeres are a hotspot for sister chromatid exchange (T-SCE). Any biological consequence of this form of instability remained obscure until quantitative modeling revealed a link between elevated T-SCE rates and accelerated cellular replicative senescence. This work strongly suggests that progressive telomere erosion is not the only determinant of replicative capacity; instead, T-SCE need to be considered as an independent factor controlling colony growth and senescence. Additionally high T-SCE rates have been observed in cells with deficiencies in WRN and BLM, the genes that are defective in Werner's and Bloom's syndromes, implying a connection to premature aging. In this Research Perspective we will explore some of the implications this recent work has for human health.

## Discovery of T-SCE

CO-FISH (Chromosome Orientation Fluorescence *In situ* Hybridization), a strand-specific modification of standard FISH invented in the early 1990s [1], is elegant in its simplicity and powerful in its application [2]. We began working with telomere probes early on as a means to determine the absolute 5'-to-3' direction of DNA sequences within chromosomes, a capability that would be useful for orienting contigs at a time when the human genome was being sequenced for the first time [3]. Because of the single-sided nature of CO-FISH, two (rather than four) fluorescent telomere signals were observed, and these marked the two 3' ends of chromo-somes when the C-rich telomere probe was hybridized. Occasionally we noted a telomere CO-FISH signal that appeared to be split between the chromatids. Once we convinced ourselves the split was real and not an artifact, we settled on sister chromatid exchange (SCE) as the most likely cause; these events are now referred to as telomere sister chromatid exchange (T-SCE).

## The search for meaning

A quick calculation revealed that telomeric DNA must be a hotspot for this kind of recombination. Any other biological consequence eluded us for several years until it was realized that within repetitive DNA the breakpoints within sister telomeres might be offset. An *unequal* exchange would cause one sister telomere to grow longer at the expense of the other. Additionally we surmised (incorrectly as it turned out) that during multiple exchanges occurring during colony expansion at least a few cells - the lucky winners in the exchange process - might maintain their telomeres well enough for the colony to escape senescence [4]. This recombination-based mechanism might explain alternative lengthening of telomeres (ALT), a mechanism used by some tumors to maintain telomeres in the absence of telomerase [5]. The losers - those cells inheriting the shorter sister telomeres - were placed at risk for entry into early senescence.

## Power of quantitative models

The process through which unequal T-SCE impacts colony growth, which includes many telomeres randomly engaging in SCE in each cell cycle, is far too complex to intuitively gauge its effect on cellular proliferation with any degree of confidence. To gain insight into the relationship between T-SCE and colony growth, we developed a quantitative model of cell proliferation in the presence of T-SCE to simulate clonal growth and eventual senescence of cells whose telomeres engaged in T-SCE, as well as suffered progressive telomere loss [6]. The model's only assumption is that the location of an exchange breakpoint in one sister telomere is independent of the breakpoint in the other sister telomere, thus allowing SCE to be unequal. The results (to our surprise) convincingly rejected T-SCE as a mechanism of ALT. Instead of prolonging proliferation, the computer simulations of our model demonstrated that T-SCE drove an early rise in the proportion of senescent cells. This effect, although unexpected, provided a mechanistic explanation for a correlation between accelerated replicative senescence in cells from Werner's and Bloom's syndrome patients [7], and the high T-SCE rates we observed [8].

**Figure 1.**
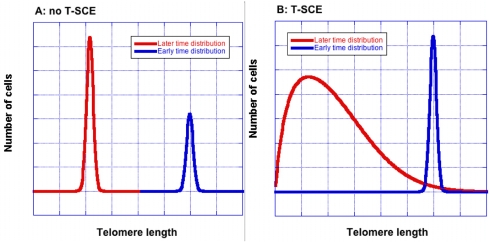
T-SCE broaden the telomere size distribution. Without T-SCE (Panel A), the shape remains unchanged as average telomere length decreases. Blue and red curves show telomere lengths at earlier and later times respectively. With T-SCE (Panel B), the distribution becomes wider with increasing cell divisions. The curves were created using an equation that was derived to allow quick, approximate calculations of T-SCE effects [9]; they are for illustration only and are not drawn to scale.

To understand how T-SCE have the potential to influence proliferation, one needs to consider the effect of T-SCE on the distribution of telomere lengths in a cell colony. By shuffling possibly unequal segments of telomeric DNA between sister telomeres, T-SCE broadens the telomere size distribution. This effect is demonstrated in Figure 1. If there were no T-SCE and each telomere lost the same amount of DNA with each cell division, the telomere size distribution retains its initial shape, while growing in size. When T-SCE occur, not only does the average length decrease with each cell division but the distribution broadens as well. As a consequence, critically short telomeres appear at earlier cell divisions and in greater numbers for the same average telomere length. The accumulation of senescent cells follows the same trend. Moreover, each cell that senesces early fails to continue its lineage; thus earlier senescence equates to smaller colony size.

If T-SCE drive early senescence, then how can ALT cells, with their high T-SCE rates, achieve unlimited colony growth? We assume ALT triggers an as yet unidentified recombination-based mechanism that adds telomeric repeats to chromosome ends. Although T-SCE are not the underlying mechanism of ALT, the short telomeres created by T-SCE may serve as a substrate for the ALT mechanism. In this case, T-SCE might promote telomere lengthening without themselves being involved in the addition of telomeric repeats. The same may apply to other instances of telomere lengthening in the absence telomerase as well [10-12].

The T-SCE effect on colony growth has only one requirement - the possibility of exchanging unequal quantities of DNA. Experimental procedures will need to be devised to measure not only the rate of exchange but also the quantities of exchanged material. These measurements would directly test the engine of the T-SCE effect. They also will reveal important details about the exchange process(es) that will help to refine the model. Additionally we recognize that DNA damage and repair in telomeric DNA may affect average telomere length, as well as promote T-SCE. In our view, the role of T-SCE does not diminish the importance of telomere shortening in replicative senescence; rather it adds a new dimension to telomere dynamics and its effect on colony growth. Quantitative modeling, together with good experimental data, will be required in order to unravel the individual contributions to replicative senescence of these, and perhaps other, telomere-related processes.

## Implications for human health

Aging is associated with a declining ability to maintain tissue structure and function. Replicative senescence contributes to aging in two ways, by limiting regenerative processes that require cell turnover and through the disruptive effects that senescent cells have on tissues [13]. These effects are likely to be amplified *in vivo* where the declining pool of replicatively competent cells would be called upon to divide more often to fulfill the needs of tissue regeneration. The result is to deplete the remaining cellular reproductive reserves at an ever accelerating pace. With these effects in mind, we argue that high T-SCE rates and their connection to accelerated replicative senescence forge a plausible mechanistic link to premature aging in Werner's and Bloom's syndromes [14].

Beyond WRN and BLM, any gene that when defective increases T-SCE should be considered as a candidate “aging” gene. A search for such genes may reveal new genetic associations with premature aging, as well as candidate genetic polymorphisms that influence the rate of normal aging. Moreover, each newly discovered T-SCE modulating gene offers yet another opportunity to test the hypothetical dependence of replicative senescence on T-SCE rate [12,15-17].

T-SCE are likely to have an impact beyond premature aging syndromes. An example is the role of ultraviolet light (UV) in skin aging [18], for which the role of telomere loss so far has been equivocal [19,20]. UV is a known inducer of SCE throughout the genome [21]. It will be informative to determine if UV exposure elevates SCE specifically within telomeres. We are currently investigating this possibility and predict that higher T-SCE rates, if observed, will be accompanied by a broader telomere size distribution and diminished replicative potential. Any broadening of the telomere size distribution is irreversible - effectively providing a “memory” of the exposure. Consequently, harmful effects such as skin aging or skin cancer brought on by a transient UV exposure in childhood, like sunburn, might not become evident until much later in life, and would be caused by a more rapid accumulation of senescent cells in skin tissue than would have occurred otherwise.

Chemicals, stress, inflammation, and poor nutrition should also be analyzed for T-SCE induction. Depending on the site of action, the effects could be localized, tissue-specific or systemic. In a preliminary study (unpublished observation), we observed reduced T-SCE in cells treated with N-acetyl-L-cysteine, suggesting that antioxidants may ameliorate some of T-SCE's harmful effects. Studies such as these will ultimately improve recommendations for healthy aging.

## Perspective

The large and growing contingent of proteins implicated at chromosome ends is a sure sign that telomeres require special attention. Many of these are DNA repair enzymes, and some have telomere-specific activities. We previously demonstrated an unanticipated role for DNA repair proteins in mammalian chromosome end protection [22]. Considering their deleterious effects, cells must have evolved effective means to carefully regulate SCE frequency in telomeric DNA. We hypothesize that nature has once again called upon DNA repair proteins and their abilities to attend to the special needs of our chromosomes' ends.
